# A Machine Learning–Guided Lyric Analysis Peer Support Intervention (LAPSI) for Psychological Distress in South African Women: Protocol for an Exploratory Sequential Mixed Methods Study

**DOI:** 10.2196/90426

**Published:** 2026-10-21

**Authors:** Lucas Banda, Mathildah Mokgatle, Olanrewaju Oladimeji

**Affiliations:** 1 Department of Public Health Faculty of Healthcare Sciences Sefako Makgatho Health Sciences University Pretoria, Gauteng South Africa; 2 Department of Social Sciences, Demography and Population Unit Faculty of Law, Humanities and Social Sciences Walter Sisulu University Mthatha, Eastern Cape South Africa; 3 Department of Public Health Faculty of Healthcare Sciences Sefako Makgatho Health Sciences University Garankuwa, Gauteng South Africa; 4 SAMRC/SMU Public Health Interventions, Innovations and Implementation Research Unit Pretoria, Gauteng South Africa

**Keywords:** anxiety, intervention, lyric analysis, machine learning, mental health, peer support, psychological distress, South Africa, stress, women

## Abstract

**Background:**

Psychological distress disproportionately affects women in South Africa and is shaped by complex structural, socioeconomic, and psychosocial determinants, particularly within the context of HIV. Despite policy commitments to integrate mental health services into existing health systems, current interventions are limited in their capacity to address these intersecting determinants and are frequently insufficiently targeted, contextually responsive, or inclusive. Data-driven strategies that identify subgroup-specific vulnerabilities may improve the design of contextually relevant and culturally grounded psychosocial interventions.

**Objective:**

This study seeks to develop and implement a machine learning (ML)–informed lyric analysis peer support intervention (LAPSI) and to evaluate its preliminary impact, feasibility, and acceptability among South African women living with or at heightened risk of HIV.

**Methods:**

An exploratory sequential mixed methods design is used across five phases: (1) a scoping review of mental health interventions in sub-Saharan Africa; (2) ML-based risk profiling using national survey data with Shapley additive explanations (SHAP)–based interpretability; (3) intervention development and implementation; (4) a single-arm pre-post quantitative assessment of psychological distress using the Kessler 10-Item Psychological Distress Scale (K10), without a comparison group; and (5) qualitative evaluation of feasibility, usability, and acceptability.

**Results:**

Secondary data analyses were conducted between October 2024 and April 2025. LAPSI was implemented between May and August 2025 in 2 provinces. Outcome assessments were completed in August 2025 (Eastern Cape) and September 2025 (Gauteng), and qualitative interviews were conducted 5 to 7 days after the intervention. A total of 30 participants were recruited and completed the intervention. Quantitative and qualitative analyses were ongoing at the time of manuscript submission, with full findings anticipated to be reported in 2026 and 2027.

**Conclusions:**

This protocol describes a potentially scalable framework for integrating explainable ML with culturally grounded, peer-delivered intervention design to address psychological distress among women in HIV-affected settings.

**International Registered Report Identifier (IRRID):**

DERR1-10.2196/90426

## Introduction

Psychological distress and prevalent mental disorders contribute substantially to the global burden of disease, accounting for more years lived with disability than numerous other leading health conditions, including both communicable and noncommunicable diseases [1]. This burden is particularly pronounced in sub-Saharan Africa, where entrenched structural inequities, constrained access to mental health services, and persistent public health challenges, including the HIV/AIDS epidemic, converge to intensify adverse mental health outcomes [1-3]. South African women experience a disproportionately high burden of psychological distress, defined as aversive mental and somatic symptoms associated with normal mood fluctuations in most people [4-7].

Despite the magnitude and complexity of this burden, mental health services in South Africa remain underresourced, fragmented, and inadequately responsive to contextually appropriate interventions. Existing programs are frequently not tailored to the lived realities of the affected populations and often neglect the need for culturally relevant, accessible, and community-based approaches [1,7-11]. In addition, a substantial proportion of existing interventions predominantly target women living with HIV, while comparatively limited attention is directed toward women who are HIV-negative, thereby underscoring a critical gap in comprehensive and inclusive mental health strategies [10,11]. There is, therefore, an urgent need to develop and implement contextually grounded interventions capable of addressing the heterogeneous, multilevel determinants of psychological distress.

Emerging empirical evidence indicates that culturally grounded, community-based approaches—such as peer-led support initiatives and music-mediated interventions—constitute promising alternative modalities for the provision of mental health support [12]. Peer-led intervention models cultivate shared experiential understanding, enhance relational trust, and improve service accessibility, whereas music-based methodologies, including structured lyric analysis, offer systematic opportunities for emotional expression, reflective processing, and the construction of personal and interpersonal meaning [13,14]. Empirical investigations, such as the Community Health Intervention Through Musical Engagement (CHIME) study, provide evidence for the efficacy and potential of music-based psychosocial interventions in comparable contexts [15]. However, the application of such approaches in South Africa, particularly in structured and standardized forms, remains limited.

While these approaches demonstrate considerable promise, a central limitation of current interventions is the lack of systematic methods for identifying and targeting the specific determinants of psychological distress across diverse population subgroups. Machine learning (ML) provides a data-driven framework for analyzing complex, multidimensional determinants and identifying risk profiles that may remain undetected by traditional analytical techniques [16,17]. By incorporating explainable ML methodologies, such as Shapley additive explanations (SHAP), it becomes feasible to derive interpretable insights into the ways in which structural, psychosocial, and health-related factors interact to influence levels of distress [18]. These insights provide a mechanism for linking population-level risk patterns to targeted intervention design.

In response to these identified gaps, this study proposes an ML-guided arts-based lyric analysis peer support intervention (LAPSI) that integrates data-driven risk stratification with culturally grounded, peer-mediated mental health support. Using a sequential mixed methods design that combines systematic evidence synthesis, predictive modeling, and intervention development, the study seeks to design, implement, and conduct a preliminary evaluation of the intervention’s impact, feasibility, and acceptability. The overarching objective is to establish a contextually responsive intervention for South African women living with or at elevated risk of HIV.

The study is aligned with South Africa’s National Strategic Plan for the Prevention and Control of Non-Communicable Diseases (2022-2027) and the National Mental Health Policy Framework and Strategic Plan (2023-2030), both of which emphasize the integration of mental health services into primary care and the development of accessible, community-based interventions for vulnerable populations, including women and people living with HIV [19,20]. More broadly, the study contributes to global priorities reflected in Sustainable Development Goals 3 (good health and well-being), 5 (gender equality), and 10 (reduced inequalities) through its focus on equitable, culturally responsive mental health support for women experiencing psychological distress [21].

**Figure 1:**
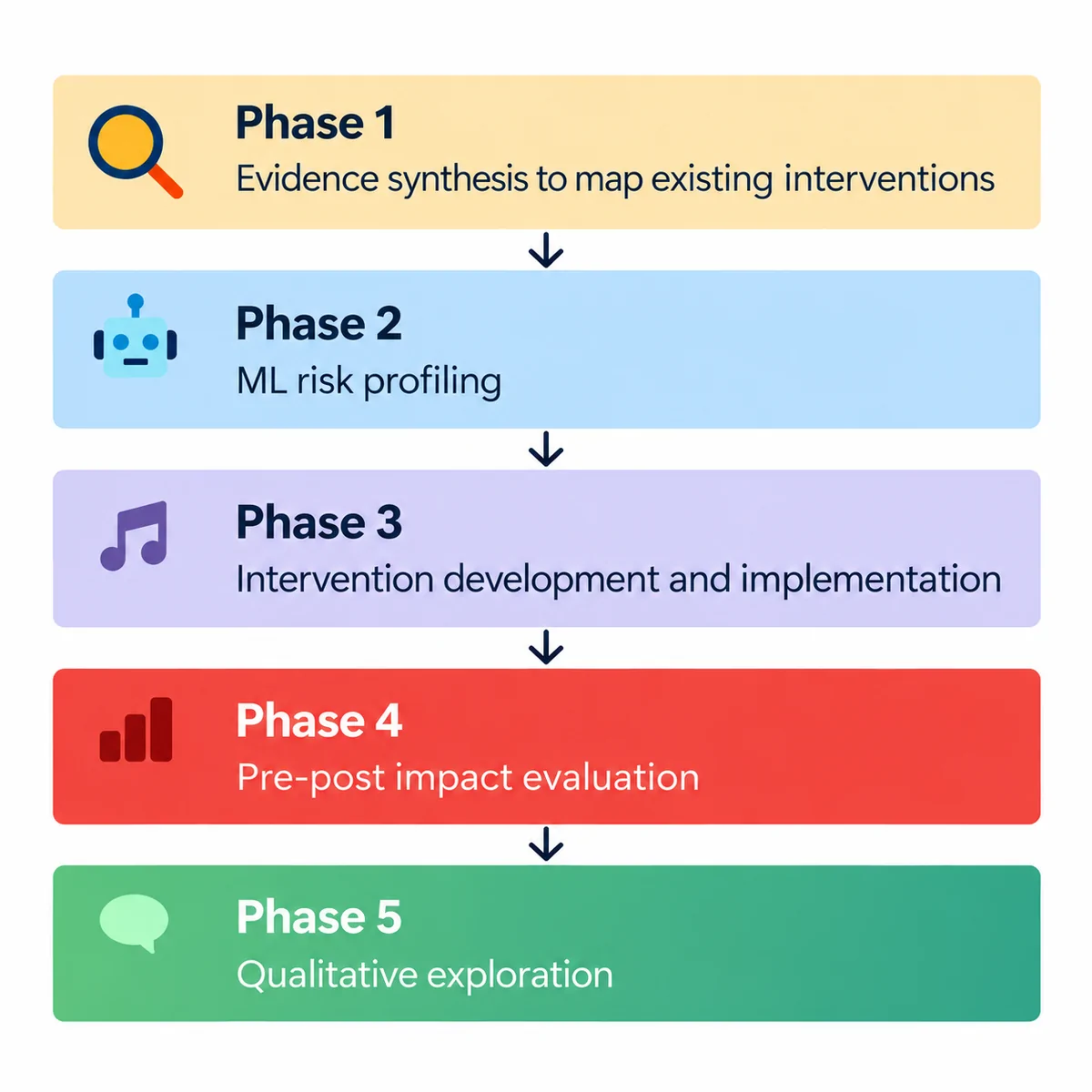
Conceptual overview of the 5 study phases. The study adopts an exploratory sequential mixed methods approach comprising evidence synthesis (phase 1), machine learning (ML)–based risk profiling (phase 2), intervention development and implementation (phase 3), quantitative pre-post evaluation of psychological distress (phase 4), and qualitative exploration of participant experiences (phase 5). Findings from the ML phase inform intervention development, while the quantitative and qualitative phases provide complementary evidence regarding feasibility, usability, acceptability, and preliminary changes associated with the lyric analysis peer support intervention (LAPSI).

## Methods

### Ethical Considerations

Ethical clearance for this study was obtained from the Sefako Makgatho Health Sciences University Research Ethics Committee (reference number SMUREC/H/305/2024:PG). Supplementary permissions were secured from the Eastern Cape Provincial, District, and Sub-District Departments of Health (reference number EC_202504_012) as well as from the Gauteng Provincial Department of Health (reference number GP_202504_055). All study procedures were conducted in accordance with institutional policies and applicable national ethical regulations and guidelines.

Written informed consent will be obtained from all adult participants prior to their enrollment in the intervention and qualitative interview components of the study. Each participant will be provided with a participant information sheet detailing the study objectives, procedures, potential risks and benefits, the voluntary nature of participation, and the right to withdraw from the study at any time without penalty or other adverse consequences. For participants aged younger than 18 years who meet the eligibility criteria, assent will be sought in conjunction with written informed consent from a parent or legal guardian, in accordance with applicable ethical guidelines and regulatory requirements.

Confidentiality will be strictly prioritized in recognition of the sensitive nature of discussions pertaining to mental health and HIV. Participant data will be anonymized by assigning unique identification codes, ensuring that no direct personal identifiers are associated with transcripts or datasets. Access to the data will be restricted to the primary researcher and designated academic supervisors, and all electronic files will be stored on password-protected systems with appropriate security safeguards.

In view of the LAPSI’s group-based structure, participants will be explicitly informed that absolute confidentiality in group sessions cannot be fully guaranteed. At the outset of each session, clear ground rules will be articulated, underscoring mutual respect and emphasizing the ethical obligation not to disclose personal or sensitive information shared by other participants outside the group context.

All quantitative and qualitative data will be stored securely on password-protected devices and institutional servers. Audio recordings will be transcribed verbatim and subsequently anonymized prior to analysis. All procedures for data management will be conducted in accordance with institutional data protection policies, and only deidentified datasets will be used for statistical analysis, interpretation, and dissemination of findings.

### Aim, Design, and Setting of the Study

This study aims to develop, implement, and evaluate an ML-guided, lyric-based peer support intervention for reducing psychological distress among South African women living with or at risk of HIV/AIDS. This study is designed as a sequential, integrated research program in which each phase directly informs the next. The scoping review (phase 1) provides an evidence base by identifying gaps in existing mental health interventions for women living with or at risk of HIV in sub-Saharan Africa. Building on this, the ML phase (phase 2) analyzes secondary data to identify context-specific predictors and risk profiles associated with psychological distress, thereby generating data-driven insights into key vulnerability factors. These findings are then translated into intervention design in phase 3, where the LAPSI is developed and implemented using themes derived from ML outputs. Phase 4 evaluates intervention-associated changes in psychological distress, providing preliminary quantitative evidence of impact, while phase 5 complements this by exploring participant experiences, feasibility, usability, and acceptability through qualitative inquiry. Together, these phases form a coherent framework that integrates evidence synthesis, predictive modeling, intervention development, and mixed methods evaluation to ensure that the intervention is both data-informed and contextually relevant.

### Phase 1: Evidence Synthesis Using Scoping Review Approach

The scoping review will follow the methodological framework proposed by Arksey and O’Malley [22] and will be reported in accordance with the PRISMA-ScR (Preferred Reporting Items for Systematic Reviews and Meta-Analyses Extension for Scoping Reviews) guidelines [23]. No formal protocol registration was undertaken for the scoping review.

A comprehensive scoping review will be conducted to examine literature published between 2014 and 2024, using electronic databases including PubMed, Scopus, and Web of Science. The review aims to identify interventions addressing psychological distress among HIV-affected women in sub-Saharan Africa.

Data from eligible studies will be extracted independently by 2 reviewers using a standardized data extraction form. This form will capture key study characteristics, including first author, year of publication, study design, study setting, sample size, and the specific types of interventions evaluated. In addition, the form will document characteristics of the study population, methodological details, and the principal findings of each study.

Drawing on the initial scoping phase, a data extraction framework will be developed to guide the retrieval and assessment of eligible full-text articles. This framework will be iteratively refined throughout the full-text review process to incorporate newly identified categories or other salient information emerging from the included studies.

Each article selected for review will be evaluated for relevance based on its publication date, which must fall between January 2014 and December 2024. This interval aligns with the First Ten-Year Implementation Plan of Agenda 2063. The selected time frame is intended to capture contemporary interventions and mental health strategies that are directly pertinent to current policy frameworks in sub-Saharan Africa. Over this period, substantial advances have been documented in mental health research as well as in the integration of mental health services within HIV care [24]. This approach will enable the examination of emerging trends and their implications for women living with and without HIV. In addition, there has been a substantial increase in research addressing mental health conditions in sub-Saharan Africa over the specified period, resulting in a more comprehensive evidence base for the present review.

Inclusion criteria are defined as follows: empirical studies involving women of reproductive age (15 to 49 years), with or without HIV infection; studies conducted in countries located within sub-Saharan Africa; evaluations of mental health interventions, including psychological, pharmacological, psychosocial, or community-based approaches; and assessment of mental health outcomes, such as depression, anxiety, posttraumatic stress disorder, psychological distress, or psychological well-being. All empirical study designs are eligible, including randomized controlled trials, quasi-experimental studies, cohort studies, cross-sectional studies, and qualitative research. Consistent with the objective of this scoping review, eligibility is restricted to English-language publications published between January 2014 and December 2024 to capture contemporary intervention strategies and current evidence gaps. Studies reporting female-specific findings, as well as mixed-gender studies in which women comprised the majority of participants and in which outcomes were clearly applicable to women, will also be included.

Exclusion criteria comprise the following: studies focusing exclusively on men, children, pregnant women, or postnatal mothers aged younger than 15 years; studies conducted outside sub-Saharan Africa; studies involving mixed-sex populations that did not provide sex-disaggregated data applicable to women; and publications classified as case reports, case series, study protocols, editorials, commentaries, or letters. Studies published prior to January 2014 were also excluded.

To enhance consistency in study selection and data extraction, 2 members of the research team will conduct a pilot test of the screening and data-charting procedures using a subset of the included studies. Based on this pilot phase, the categorization framework will be refined as necessary, particularly in instances where new categories emerge. Any discrepancies between reviewers will be addressed through discussion within the research team and, when required, by consulting a third reviewer. In cases where abstracts meet the inclusion criteria but provide insufficient information, the corresponding authors will be contacted to obtain the missing data.

Primary responsibility for data charting for each included study will be assigned to the team members who participated in piloting the data extraction framework. Any discrepancies in extracted data between these 2 reviewers will be resolved through iterative discussion until consensus is reached or, if necessary, through adjudication by a third reviewer.

A comprehensive and reproducible search strategy will be developed using predefined descriptors in combination with Boolean operators. The search terms will encompass the following four core conceptual domains: (1) mental health interventions (eg, psychological, pharmacological, psychosocial, and community-based approaches), (2) mental health outcomes (eg, psychological distress, depression, anxiety, posttraumatic stress disorder, and well-being), (3) HIV status (including women living with and without HIV), and (4) geographic context (sub-Saharan Africa). Database-specific search syntax will be applied across PubMed, Scopus, Web of Science, and PsycINFO. Complete search strings and the corresponding Boolean logic are presented in Multimedia Appendix 1.

### Phase 2: ML-Guided Risk Profiling

To identify predictors of psychological distress, 6 supervised ML algorithms will be systematically evaluated and compared: logistic regression, decision tree, random forest, K-nearest neighbors (KNN), gradient boosting, and extreme gradient boosting (XGBoost). These algorithms were selected because they encompass a spectrum of methodological paradigms, including traditional statistical learning, tree-based models, ensemble techniques, and distance-based classifiers, all of which are widely applied in predictive modeling within health research.

Model performance will be quantified using multiple evaluation metrics, namely accuracy, precision, recall, *F*_1_-score, and the area under the receiver operating characteristic curve (AUC-ROC), to provide a comprehensive assessment of discriminative ability and classification quality. The final model will be chosen based on an integrated appraisal of overall predictive performance, robustness across validation procedures, and degree of interpretability.

Special emphasis will be placed on model explainability to support the practical translation of identified risk factors into targeted intervention strategies. To this end, SHAP analysis will be used to decompose model predictions into feature-level contributions, thereby elucidating the relative importance and directionality of individual predictors of psychological distress.

To operationalize ML outputs within the LAPSI intervention, predictors identified via SHAP will be systematically mapped to thematic domains that structure session content. Predictors demonstrating consistent importance across models, such as employment status, HIV-related stigma, health care access, and perceived health, will be aggregated into higher-order psychosocial and structural categories. Each category will subsequently be mapped onto a corresponding intervention theme (eg, socioeconomic stress, stigma, health care access, or wellness and self-assessment), which will guide the content, emphasis, and sequencing of individual sessions.

These themes will be operationalized through lyric-analysis-based discussions, in which carefully selected songs that reflect each thematic domain serve as prompts to facilitate guided reflection, emotional processing, and peer interaction. In this manner, ML-derived risk profiles will not be limited to predictive functions; rather, they will be translated into structured, contextually salient intervention components that directly address the determinants of psychological distress identified in phase 2.

In this phase of the study, only data pertaining to adults aged 15 to 49 years will be used, as this group constitutes the target population. Specifically, the analysis will be restricted to women aged 15 to 49 years who are living with HIV or are at risk of HIV infection, as identified within the secondary dataset. All males, as well as females aged younger than 15 years or older than 49 years, will be excluded from the analytical sample. This study uses secondary data analysis and ML methodologies to develop risk profiles and identify predictors of psychological distress among women living with HIV, as well as women at elevated risk of HIV acquisition, in South Africa.

Data for this investigation will be drawn from the 2012 and 2017 waves of the South African National HIV Prevalence, Incidence, Behaviour and Communication Survey (SABSSM [25]; Zuma et al [26]). The SABSSM dataset encompasses several demographic strata, including guardians (aged 0-11 years), children (aged 12-14 years), and adults (aged 15 years and older). Permission to access and use the SABSSM data was obtained from the Human Sciences Research Council (HSRC). The data access application, which included a summary of the research protocol, was submitted to the HSRC and received ethical and administrative approval following a brief review period. The survey methodology implemented in the 2017 study used a multistage, disproportionate, stratified cluster sampling design, consistent with the approach used in earlier surveys. In this iteration, however, the primary sampling unit (PSU) was redefined: small area layers (SALs) replaced the enumeration areas (EAs) that had previously served as PSUs in earlier survey rounds. A new master sample was constructed through disproportionate stratified sampling of 1000 SALs drawn from the 2011 National Sampling Frame, which consists of 84,907 SALs as delineated by Statistics South Africa. Selection of SALs was based on probability proportional to size, with the number of vulnerable populations (VPs) within each SAL used as the measure of size. The selected SALs were updated in 2015 and geospatially delineated using aerial imagery to generate a revised master sample. This master sample formed the basis for household selection in the 2017 survey [25,26].

The primary outcome variable in this study is psychological distress, operationalized in 2 ways. First, it will be treated as a continuous variable using the Kessler 10-Item Psychological Distress Scale (K10). Individual item scores on the K10 will be summed to produce a total score, which is then used to classify respondents into categories of mental distress. A total score of less than 20 indicates probable psychological well-being, whereas scores of 20 to 24, 25 to 29, and 30 or higher denote mild, moderate, and severe psychological distress, respectively.

For additional analyses, the K10 scores will be dichotomized to facilitate clinically meaningful classification and to support interpretation and comparison of proportions over time. Specifically, scores below 20 will be coded as 0 (no distress), and scores of 20 or higher will be coded as 1 (distress). Although dichotomization may entail some loss of information, both continuous and binary representations of the K10 will be analyzed to provide a more comprehensive evaluation of changes in psychological distress.

Several independent variables will be incorporated in this study. Table 1 summarizes the types of variables (features), their labels, and the procedures that are used for their coding and measurement. Biological variables include HIV serostatus. HIV serostatus is operationalized as a binary variable, whereby individuals who test HIV-negative are assigned a value of 0, and individuals who test HIV-positive are assigned a value of 1.

**Table 1:** Types of features, names of features, and coding and measurement of outcome and predictor variables used for machine learning–based risk profiling of psychological distress among South African women aged 15 to 49 years.

Type of feature and feature name					Measurement and coding
Output/dependent					
	Psychological distress			Continuous measure: scores less than 20 indicate likely psychological well-being, scores of 20 to 24 indicate mild distress, scores of 25 to 29 indicate moderate distress, and scores of 30 or greater indicate severe distress. Binary measure: scores less than 20 will be coded as 0 (no distress), and scores of 20 or higher will be coded as 1 (distress).	
Independent					
	Biological				
		HIV status	HIV serostatus is coded as a binary variable, with HIV-negative individuals assigned a value of 0 and HIV-positive individuals assigned a value of 1.		
	Demographic				
		Age	Denotes the age categories of individuals aged 15 to 49 years within the dataset, encoded as follows: 15 to 19 years=0, 20 to 24 years=1, 25 to 29 years=2, 30 to 34 years=3, 35 to 39 years=4, 40 to 44 years=5, and 45 to 49 years=6.		
		Residence	Rural areas are coded as 0, and urban areas are coded as 1.		
		District	The study area encompassed 52 districts and metropolitan municipalities, namely: Uthungulu, West Coast, Cape Winelands, Overberg, Eden, Central Karoo, City of Cape Town, Buffalo City, Cacadu, Amathole, Chris Hani, Joe Gqabi, O.R. Tambo, Alfred Nzo, Nelson Mandela Bay, John Taolo Gaetsewe, Pixley ka Seme, Siyanda, Frances Baard, Xhariep, Lejweleputswa, Thabo Mofutsanyane, Fezile Dabi, Mangaung, Ugu, Uthukela, Amajuba, Zululand, iLembe, uMgungundlovu, Umzinyathi, Umkhanyakude, Sisonke, eThekwini, Bojanala, Ngaka Modiri Molema, Dr Ruth Segomotsi Mompati, Dr Kenneth Kaunda, Sedibeng, West Rand, Ekurhuleni, City of Johannesburg, City of Tshwane, Gert Sibande, Nkangala, Ehlanzeni, Mopani, Vhembe, Capricorn, Waterberg, Greater Sekhukhune, and Namakwa.		
		Province	The following numerical identifiers were assigned to the nine provinces of South Africa: Eastern Cape (1), Free State (2), Gauteng (3), KwaZulu-Natal (4), Limpopo (5), Mpumalanga (6), North West (7), Northern Cape (8), and Western Cape (9).		
		Race	Ethnic group categories were operationalized using the following numerical coding scheme: African (0), White (1), Colored (2), and Indian/Asian (3).		
	Socioeconomic				
		Marital status	Unmarried was coded as 0, and married was coded as 1.		
		Employment status	Employment status is coded as follows: unemployed (actively seeking employment)=0, employed=1, and not in the labor force=2.		
		Education level	Secondary education (grades 8-11) is coded as 0, primary education (grades R-7) is coded as 1, matriculation (grade 12) is coded as 2, and postmatric (tertiary or higher) education is coded as 3.		
	Environmental				
		HIV stigma	Binary responses were coded as follows: no=0 and yes=1.		
		Health care access	Categories for health care access were operationalized using the following numerical codes: other health care sources=0, government hospital=1, mobile clinic=2, pharmacy=3, private doctor=4, and private hospital or clinic=5.		

Demographic variables were operationalized as follows: age denotes the categorized age of respondents ranging from 15 to 49 years and was treated as an ordinal variable with the following coding scheme: 15 to 19 years=0, 20 to 24 years=1, 25 to 29 years=2, 30 to 34 years=3, 35 to 39 years=4, 40 to 44 years=5, and 45 to 49 years=6. Place of residence was coded as a dichotomous variable, with rural residence assigned a value of 0 and urban residence assigned a value of 1. The study area comprised 52 districts and metropolitan municipalities, specifically: Uthungulu, West Coast, Cape Winelands, Overberg, Eden, Central Karoo, City of Cape Town, Buffalo City, Cacadu, Amathole, Chris Hani, Joe Gqabi, O.R. Tambo, Alfred Nzo, Nelson Mandela Bay, John Taolo Gaetsewe, Pixley ka Seme, Siyanda, Frances Baard, Xhariep, Lejweleputswa, Thabo Mofutsanyane, Fezile Dabi, Mangaung, Ugu, Uthukela, Amajuba, Zululand, iLembe, uMgungundlovu, Umzinyathi, Umkhanyakude, Sisonke, eThekwini, Bojanala, Ngaka Modiri Molema, Dr Ruth Segomotsi Mompati, Dr Kenneth Kaunda, Sedibeng, West Rand, Ekurhuleni, City of Johannesburg, City of Tshwane, Gert Sibande, Nkangala, Ehlanzeni, Mopani, Vhembe, Capricorn, Waterberg, Greater Sekhukhune, and Namakwa. The variable province denotes the following numerical identifiers assigned to the nine provinces of South Africa: Eastern Cape (1), Free State (2), Gauteng (3), KwaZulu-Natal (4), Limpopo (5), Mpumalanga (6), North West (7), Northern Cape (8), and Western Cape (9). Racial group membership was operationalized using the following categorical coding scheme: African (0), White (1), Colored (2), and Indian/Asian (3).

Socioeconomic variables were operationalized as follows. Marital status was dichotomously coded, with unmarried participants assigned 0 and married participants assigned 1. Employment status was categorized into 3 groups: unemployed (actively seeking employment), coded as 0; employed, coded as 1; and not in the labor force, coded as 2. Educational attainment was classified according to the highest level of formal education completed: secondary education (grades 8-11), coded as 0; primary education (grades R-7), coded as 1; matriculation (grade 12), coded as 2; and postmatric (tertiary or higher) education, coded as 3.

### Environmental Variables

To derive a composite HIV stigma index, responses to the relevant items are aggregated into a summed scale. Scores below the sample mean are interpreted as reflecting comparatively low levels of stigma, whereas scores above the sample mean are interpreted as reflecting comparatively high levels of stigma. This continuous index is subsequently dichotomized, with low stigma coded as “no” (0) and high stigma coded as “yes” (1). The internal consistency reliability of the scale will be assessed using Cronbach α. An α coefficient of 0.60 or higher will be deemed acceptable, and preliminary analyses indicate an α coefficient of 0.70.

Health care access: categories of health care access were operationalized using the following numerical codes: other health care sources=0, government hospital=1, mobile clinic=2, pharmacy=3, private doctor=4, and private hospital or clinic=5.

Model development will use a stratified train-test split, allocating 70% of observations to the training set and reserving 30% for the test set to obtain approximately unbiased estimates of out-of-sample performance. Within the training partition, stratified k-fold cross-validation will be implemented to optimize model hyperparameters and to mitigate overfitting. Class imbalance in the binary psychological distress outcome will be addressed using the synthetic minority oversampling technique (SMOTE), which will be applied exclusively within the training folds to avoid information leakage into the validation or test data.

Model performance will be quantified using a suite of complementary evaluation metrics, including accuracy, sensitivity (recall), specificity, precision (positive predictive value), *F*_1_-score, and the ROC-AUC. To enhance interpretability and support ethical transparency, SHAP will be used to estimate the contribution of individual predictors at both the global (model-level) and local (individual-level) scales. Predictors demonstrating consistent and robust importance across models will be aggregated into higher-order thematic domains to guide the conceptualization, design, and content development of the LAPSI.

### Phase 3: Development and Implementation of the LAPSI Intervention

In phase 3, the LAPSI will be developed and implemented as a contextually tailored, culturally grounded mental health intervention specifically targeting women living with or at elevated risk for HIV who are experiencing psychological distress. The intervention is theoretically and empirically informed by evidence indicating that peer support facilitates emotional safety, mutual recognition through shared experiences, and enhancement of psychological empowerment [12], while systematic lyric analysis promotes reflective dialogue and facilitates coping processes through music that is embedded in culturally familiar contexts [13,14]. LAPSI seeks to integrate these strengths by implementing a lyric-informed peer support framework that is explicitly calibrated to the individualized risk profiles derived from ML models developed in phase 2.

### Sampling and Sample Size

#### Overview

The study will use convenience sampling, a nonprobability sampling technique that is widely used in exploratory and resource-constrained research, particularly within quasi-experimental designs, wherein pragmatic considerations frequently take precedence over stringent sampling rigor [27,28]. In low-resource settings, challenges such as financial constraints may limit the feasibility of recruiting large samples, while the sensitive nature of the research topic may further reduce participation by making potential participants reluctant to engage. The planned sample size of approximately 30 participants was considered appropriate for this exploratory feasibility study and aligns with recommendations for pilot and feasibility research, which emphasize the assessment of feasibility, acceptability, implementation processes, and preliminary outcome patterns rather than definitive tests of intervention effectiveness [29,30]. The sample size was also considered sufficient to support the study’s mixed methods objectives, including pre-post quantitative assessment and in-depth qualitative exploration of participants’ experiences.

#### Recruitment Approach

To address these challenges, the recruitment strategy will be adapted to the specific context of each clinic. In some clinics, telephonic recruitment will be used, whereby existing clinic records will be systematically reviewed to identify potential participants who meet preliminary criteria. These individuals will then be contacted by telephone and invited to take part in the study. In other clinics, printed pamphlets will be distributed and placed in waiting areas. Interested individuals will be invited to contact the research team—preferably via WhatsApp—to indicate their willingness to be considered for participation. Subsequently, these individuals will receive a follow-up telephone call during which eligibility will be formally assessed, and those who meet the inclusion criteria will be invited to enroll in the study. These recruitment procedures are aligned with and informed by prior peer-reviewed investigations that have implemented comparable methodological strategies [31,32]. These strategies fall within the broader category of nonprobability sampling, as participants are recruited on the basis of accessibility and self-selection rather than through procedures of random selection [33].

#### Use of Incentives

To minimize financial barriers to participation, modest incentives were provided in the form of transport reimbursement (ZAR 100; [ZAR 1=US $0.055-US $0.056 as of May-August 2025]) and refreshments (approximately ZAR 50) during intervention sessions. These measures were designed to compensate for direct expenses incurred through participation rather than to function as coercive inducements. The level of reimbursement was deliberately kept low and applied uniformly across all participants, in alignment with ethical guidelines for research involving economically vulnerable populations, wherein incentives are intended to enable participation without exerting undue influence on decision-making [31,32].

### Processes and Intervention

The intervention will be conducted in 2 South African locations: Mthatha (Eastern Cape) and Soshanguve (Gauteng Province). These sites have been purposefully selected based on epidemiological evidence of elevated HIV prevalence and documented mental health disparities affecting women in both provinces [25,34]. Only a single facilitator will be used in the study. The use of a single facilitator across all sites is intended to enhance consistency in intervention delivery, while the research assistant received standardized orientation regarding study procedures, participant support, ethical conduct, and intervention implementation. The second phase of the study advances the third research objective by translating the identified data-informed risk profiles into targeted participant groupings and structured peer-support sessions that use lyric analysis as a systematic technique for emotional exploration. Stratified peer groups, comprising a minimum of 3 and a maximum of 12 participants, are guided through a series of 30- to 45-minute sessions, each focused on a single SHAP-informed theme anchored in selected songs nominated by participants or facilitators.

To operationalize the ML outputs into intervention content, SHAP-identified predictors will be synthesized into overarching thematic domains, which will subsequently be mapped onto specific session topics. Song lyrics exemplifying these themes will serve as structured prompts for group discussion, thereby enabling participants to articulate, examine, and contextualize their own experiences. This design ensures that the intervention content is directly derived from empirically identified risk factors, rather than from generic or a priori assumptions.

The intervention will be implemented either as a single-day intensive workshop or in a multisession format spread over several weeks, contingent on site-specific logistical constraints. Although the delivery format may differ, the total number of sessions, overarching thematic structure, pedagogical activities, and content sequence will be standardized across all sites. This delivery flexibility is not expected to compromise reproducibility. The implementation strategy is consistent with established methodological guidance for complex interventions, which advocates preserving core components while permitting adaptations in mode of delivery to optimize feasibility and contextual appropriateness [35,36]. This consideration is particularly salient in community-based settings, where rigid implementation schedules may be impractical. To preserve reproducibility under these conditions, a standardized intervention protocol was used in conjunction with a structured session guide specifying predefined themes, activities, and sequencing across all sites. Intervention fidelity was supported through adherence to the intervention manual, use of standardized session procedures, and the delivery of all sessions by the primary researcher. This approach was intended to ensure that sessions were delivered within a uniform framework, thereby promoting consistency in both content and facilitation across study sites. Lyric discussion will be organized according to a 5-stage processing model [13,37] developed to systematically scaffold participant engagement in meaning-making processes. This framework provides a structured approach to the analysis of song lyrics in therapeutic group contexts, with each stage supporting progressive emotional exploration and self-reflection, moving from initial, surface-level engagement toward more complex personal insight and functional application.

In the foundation stage, participants focus on literal comprehension of the lyrical content. The reflection stage emphasizes the identification and articulation of emotional responses. The group process stage promotes shared resonance and collective interpretation within the group context. The personal insight stage facilitates individualized meaning-making and the integration of lyrical themes with participants’ own experiences. Finally, the transfer stage supports the application of emergent understandings and insights to participants’ everyday lives (Figure 2 [13,37]). This model constitutes the conceptual backbone of the intervention, providing both structural coherence and cultural responsiveness in the design and implementation of group sessions.

**Figure 2:**
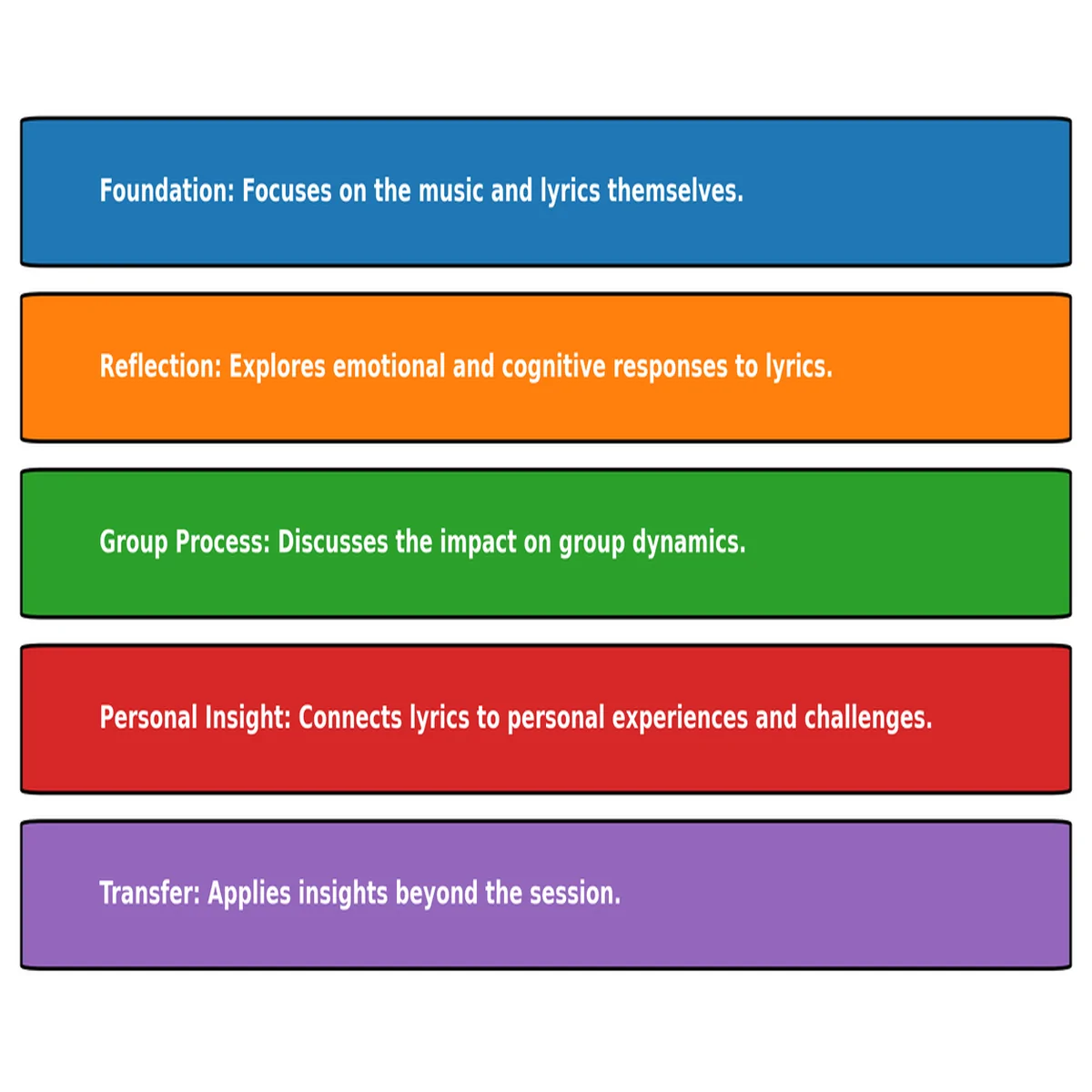
The 5-stage lyric analysis framework (Kees and Jacobs [35] as adapted by Dvorak [13]).

### Participants’ Safety

Songs will either be nominated by participants or selected from a preestablished, curated repertoire that has been used in prior empirical studies. This curated list will be reviewed collaboratively with participants, who will then determine their preferred song, either by selecting 1 piece per thematic category from the list or by engaging in group deliberation to identify a song of collective interest. All song lyrics will be systematically screened for linguistic accessibility, emotional safety, and cultural relevance and resonance.

Across all sessions, structured grounding techniques and standardized check-in prompts will be implemented to support psychological containment and mitigate emotional distress. Postsession debriefing procedures will be conducted to facilitate emotional processing and closure.

Where financial and institutional resources permit, a licensed psychologist will be present during the intervention to provide in vivo psychological support to participants who may experience significant emotional activation or distress in response to the discussion. In settings where such resources are unavailable and an on-site psychologist cannot be secured, formal arrangements will be established with the South African Depression and Anxiety Group (SADAG). In these instances, the researcher will ensure that participants have access to the necessary means (eg, telephone facilities and contact information) to engage with SADAG during the intervention, thereby enabling timely psychological and emotional support for individuals who exhibit signs of distress.

### Phase 4: Evaluation of Intervention Effectiveness

Given the absence of a control group, phase 4 is designed to examine preliminary within-subject change over time rather than to permit strong causal inference regarding intervention effects. Accordingly, findings will be interpreted as indicative of intervention-associated change and feasibility, in line with the aims of early-phase, exploratory intervention research.

Phase 4 will evaluate temporal changes in psychological distress associated with participation in the LAPSI, using a quasi-experimental, single-group pre-post design. All participants allocated to the intervention will complete the K10 at 2 time points: baseline (T0) and postintervention (T1). Because the K10 will serve as the primary outcome measure, the postintervention assessment will be administered 30 days following completion of the intervention to capture short-term impact.

The K10 has previously demonstrated robust psychometric properties in South African populations, including high internal consistency (Cronbach α=0.84) and strong reliability as indicated by omega total (ω=0.88), as well as evidence of factorial validity [38].

Variation in psychological distress across the intervention period will be assessed using scores on the K10. Although the K10 yields a continuous score ranging from 10 to 50, scores will also be categorized according to standard severity thresholds: low or no distress (<20), mild distress (20-24), moderate distress (25-29), and severe distress (≥30). For participant eligibility and binary classification analyses, scores below 20 will be classified as “not distressed” (0), whereas scores of 20 or greater will be classified as “distressed” (1). This dichotomization facilitates classification analyses and model harmonization [8,39].

### Analysis

Descriptive statistics will be used to summarize participant sociodemographic characteristics, psychosocial indicators, and both continuous and dichotomized K10 scores. These summaries will be stratified by key variables, including geographic site (Eastern Cape vs Gauteng), HIV status, and other relevant participant characteristics, to contextualize the composition of the study sample.

Given the feasibility-oriented nature of the study and the limited sample size, inferential analyses will be conducted on the full sample without stratification. Although contextual factors such as geographic site, HIV status, intervention delivery format, and baseline distress severity are recognized as potentially important sources of heterogeneity, the study is not powered to detect subgroup differences. Accordingly, these variables will be incorporated into the interpretation of results rather than examined through formal subgroup analyses. This analytic approach is consistent with feasibility and pilot study methodology, where the primary aim is to describe overall patterns of change and generate preliminary estimates to inform the design of future, adequately powered studies, rather than to support definitive subgroup inference [35,36].

Longitudinal changes in psychological distress will be examined using pooled inferential analyses. Changes in continuous K10 scores between baseline (T0) and postintervention (T1) will be evaluated using the Wilcoxon signed rank test, a nonparametric procedure appropriate for paired ordinal or continuous data that deviate from normality. Changes in dichotomous distress classification will be assessed using the McNemar test, which is suitable for paired categorical outcomes. Effect sizes (eg, Cohen *r* or matched-pairs *d*) will accompany all inferential analyses to facilitate interpretation of the magnitude of change.

### Phase 5: Qualitative Evaluation of LAPSI Acceptability and Implementation

The qualitative component of this study is structured as an exploratory evaluation aimed at assessing the feasibility, acceptability, and usability of the LAPSI among South African women living with or at elevated risk for HIV who experience psychological distress. Consistent with the exploratory orientation of feasibility-phase intervention research, no a priori hypotheses or propositions are formulated for the qualitative component.

Specifically, the qualitative evaluation will explore the following domains:

- Barriers and feasibility: the study will explore participant-perceived environmental, individual, and health‑system factors that influence engagement with and sustained participation in LAPSI, including access to services, stigma, awareness of peer‑support interventions, and contextual constraints affecting implementation feasibility.
- Facilitators and motivators: the qualitative analysis will examine participant-identified facilitators and motivators for LAPSI acceptability and uptake, including experiences of peer support, cultural resonance of lyric analysis, perceived psychological benefits, and alignment with community-based mental health approaches.
- Usability and acceptability: the study will assess the usability of LAPSI by exploring participants’ perceived ease of engagement with lyric analysis components, clarity of peer-support processes, and adaptability of the intervention across diverse literacy levels, technological access, and cultural contexts.

Findings from this qualitative component are intended to inform refinement, contextual adaptation, and future scale-up of LAPSI rather than to confirm predefined expectations.

### Interview Sample and Data Collection

The final phase of the study will use qualitative inquiry to assess participants’ experiences with the LAPSI intervention, focusing on perceived barriers, motivators, feasibility, and usability. This is critical for understanding how the intervention is received and how it might be adapted or scaled in comparable settings.

In this study, participants will be classified as either HIV-positive or HIV-negative and further segmented according to their locations in Mthatha (Eastern Cape) and Tshwane (Gauteng). Data saturation will be assessed iteratively during analysis. However, given the anticipated heterogeneity across HIV status groups and geographic sites, additional contextual insights may continue to emerge even after recurring themes are identified. Consequently, all intervention participants will be invited to participate in the qualitative component to ensure sufficient depth, breadth, and variation in experiences of the intervention. This approach is consistent with the concept of information power, which suggests that sample adequacy depends not only on the number of participants but also on the relevance, richness, and diversity of the information they can provide [40,41]. In applied intervention research, capturing diverse perspectives may be prioritized alongside, rather than being limited strictly by, saturation thresholds [40,41]. Including all participants is therefore expected to enhance the credibility and transferability of the findings by ensuring that a broad range of experiences, viewpoints, and contextual influences are represented.

Semistructured interviews will be conducted shortly following completion of the intervention. LAPSI may be administered either as a single-day intensive program or as a multisession format delivered weekly across several weeks; in both cases, interviews will be scheduled within 2 to 7 days after the final session. This timing was selected to provide participants with a brief period following completion of the intervention while ensuring that experiences remained relatively recent and accessible for reflection.

The use of qualitative exit interviews shortly after completion of clinical trials and intervention studies is consistent with approaches described in patient-centered outcomes research. Guidance from the International Society for Pharmacoeconomics and Outcomes Research (ISPOR) characterizes exit interviews as semistructured qualitative interviews conducted at or following the final study visit to capture participants’ experiences of study participation and treatment (Gater et al [42] and DiBenedetti [43]). Consistent with this approach, interviews in the present study were scheduled within 2 to 7 days of intervention completion as a study-specific decision intended to balance opportunities for participant reflection with the collection of experiences that remained recent and salient.

Interviews will be conducted in participants’ preferred language (isiXhosa, Setswana, or English), following a semistructured interview guide designed to elicit information on: (1) motivations for study participation, (2) perceived utility of lyric analysis and peer-support components, (3) barriers to engagement with the intervention, and (4) overall usability and cultural acceptability of the intervention. With participants’ informed consent, all interviews will be audio-recorded, transcribed verbatim, translated where necessary, and subsequently deidentified prior to analysis.

### Data Analysis

Thematic analysis will be undertaken using either MAXQDA or NVivo, contingent upon institutional access and licensing arrangements at the time of analysis. Both software packages facilitate systematic coding, memo-writing, maintenance of audit trails, and structured codebook management, and they are widely used for thematic analysis in public health research. The selection of software will not influence the analytic strategy, which is grounded in the 6-phase thematic analysis framework by Braun and Clarke [44]. Analytic consistency will be maintained through the application of a predefined coding framework, reflexive memo-writing practices, and iterative code review procedures, irrespective of the software platform used.

### Trustworthiness

Trustworthiness was ensured using established criteria for qualitative methodological rigor. Credibility was enhanced through triangulation across HIV serostatus and study sites, member checking with a purposive subset of participants, and regular supervisory debriefing meetings. Dependability was supported by transparent documentation of analytic decision-making processes and strict adherence to a standardized interview protocol. Confirmability was strengthened through anonymized data management procedures, systematic reflexive memo-writing, and iterative, team-based coding. Transferability was facilitated by providing rich, thick descriptions of the study contexts, participant characteristics, and analytic interpretations [45,46].

## Results

At the time of manuscript submission, secondary data analyses had been conducted between October 2024 and April 2025. The LAPSI intervention was implemented between May and August 2025 in the Eastern Cape and Gauteng provinces. A total of 30 participants were enrolled, and outcome assessments were completed in August 2025 (Eastern Cape) and September 2025 (Gauteng). Qualitative interviews were conducted 5 to 7 days after completion of the intervention. Quantitative and qualitative data analyses were ongoing at the time of manuscript submission, with comprehensive findings expected to be reported in 2026 and 2027.

## Discussion

### Expected Contribution and Implications

This study is expected to generate novel empirical evidence regarding the feasibility and potential impact of a culturally grounded, arts-based mental health intervention designed for South African women living with or at elevated risk of HIV infection. By integrating evidence synthesis, ML-driven risk profiling, and systematic intervention development within a single research program, the study aims to advance the emerging body of scholarship on gender-responsive mental health strategies in low- and middle-income country contexts. The anticipated findings may inform policy initiatives focused on the integration of mental health services into HIV prevention and treatment programs and provide an evidence base for scalable, community-based psychosocial interventions that are responsive to the needs of women experiencing psychological distress.

### Principal Anticipated Findings

The study is anticipated to yield nuanced insights into the multifaceted interplay of structural, psychosocial, and health-related determinants of psychological distress among South African women living with or at elevated risk for HIV infection. The ML component is expected to delineate both shared and context-specific risk profiles, thereby informing the development of more precisely targeted and inclusive intervention strategies. Furthermore, the intervention evaluation is intended to generate empirical evidence on the feasibility, usability, and acceptability of LAPSI as a culturally grounded, peer-delivered psychosocial intervention for women experiencing psychological distress.

### Contribution to Existing Literature and Comparison With Prior Work

Existing literature has consistently demonstrated that psychological distress among women in sub-Saharan Africa is shaped by socioeconomic adversity, stigma, gender-based violence, and constrained access to health services [47-50]. Nonetheless, interventions targeting these determinants have predominantly focused on women living with HIV, while comparatively limited attention has been devoted to HIV-negative women who experience similar structural and psychosocial vulnerabilities [51,52]. The scoping review informing this protocol identified this imbalance and underscored the need for more inclusive strategies in the design of mental health interventions.

The present study builds on previous work by integrating explainable ML with culturally grounded intervention development. Although ML approaches have increasingly been used in mental health research to identify risk factors and predict outcomes [16,53], there remains a paucity of studies that systematically translate these predictive insights into concrete intervention components. By using SHAP-based interpretation to derive interpretable risk profiles and to directly map these profiles onto intervention themes, this study moves beyond purely predictive modeling toward generating actionable, intervention-relevant insights. In doing so, it aligns with and extends existing research on peer-support and music-based interventions [12,14,15] by introducing a systematic, data-informed framework for tailoring intervention content to empirically identified determinants of psychological distress.

### Strengths of the Study

A principal strength of this protocol is its integrated, sequential design, which systematically links evidence synthesis, ML-based risk profiling, intervention development, and mixed methods evaluation within a single, coherent methodological framework. The application of explainable ML enhances analytic transparency and interpretability, thereby addressing common critiques of opaque “black-box” approaches. Furthermore, the emphasis on culturally grounded, peer-delivered intervention strategies directly responds to well-documented barriers to mental health care access and acceptability within South African settings. The inclusion of both HIV-positive and HIV-negative women constitutes an additional strength, rectifying a previously identified gap in the literature and promoting a more equitable approach to mental health intervention research. Finally, the mixed methods evaluation design facilitates the assessment of both preliminary quantitative outcomes and qualitative experiences, yielding complementary evidence regarding feasibility, usability, and acceptability.

### Limitations

Several limitations should be acknowledged. First, the pre-post design without a control group constrains the capacity to infer causal effectiveness; consequently, results will be interpreted as intervention-associated change rather than as definitive evidence of impact. Second, the feasibility-oriented sample size limits the power to conduct inferential subgroup analyses by site, HIV status, or delivery format. These issues will be managed through descriptive stratification and contextual interpretation rather than formal hypothesis testing. Finally, variability in intervention delivery format (eg, single-day vs multisession implementation) may introduce heterogeneity in participant experiences, although adherence to a standardized core intervention protocol is intended to attenuate this concern.

### Implications and Future Directions

Findings from this study are anticipated to guide the refinement and scale-up of LAPSI and to contribute to the expanding empirical evidence base for data-driven, culturally responsive mental health interventions. Subsequent research should use controlled or randomized study designs with larger and more heterogeneous samples to rigorously evaluate intervention effectiveness and implementation outcomes across diverse contexts. Furthermore, the ML-guided framework developed herein may be adapted for other populations and mental health conditions, providing a transferable model for the integration of predictive analytics into the design and optimization of mental health interventions.

### Dissemination

Results from this study will be disseminated through peer-reviewed scholarly publications, conference presentations, and engagement with community and health system stakeholders. Findings will be shared with participating communities and relevant policy actors to support the integration of evidence-based, community-driven mental health interventions within existing health and social care structures.

## Appendix

Multimedia Appendix 1 Complete search strings and the corresponding boolean logic for scoping review.

## Abbreviations

AUC-ROC: area under the receiver operating characteristic curve
CHIME: Community Health Intervention Through Musical Engagement
EA: enumeration area
HSRC: Human Sciences Research Council
K10: Kessler 10-Item Psychological Distress Scale
KNN: K-nearest neighbors
LAPSI: lyric analysis peer support intervention
ML: machine learning
PRISMA-ScR: Preferred Reporting Items for Systematic Reviews and Meta-Analyses Extension for Scoping Reviews
PSU: primary sampling unit
SABSSM: South African National HIV Prevalence, Incidence, Behaviour and Communication Survey
SADAG: South African Depression and Anxiety Group
SAL: small area layer
SMOTE: synthetic minority oversampling technique
VP: vulnerable population
XGBoost: extreme gradient boosting

## Data Availability

This study will use both secondary data from the South African National HIV Prevalence, Incidence, Behaviour and Communication Survey (SABSSM) and primary data collected from participants through fieldwork. Access to the SABSSM dataset is subject to the conditions and agreements set by the Human Sciences Research Council (HSRC) and cannot be publicly shared. The primary qualitative and quantitative data collected during this study will be accessible only to the principal investigator, coinvestigators, research supervisors, and the affiliated academic institution, in accordance with ethical approval and participant consent. Per institutional guidelines, all raw data will be securely stored and destroyed 5 years after completion of the study. Only deidentified, aggregated results will be made available within the manuscript and supplemental materials.

## Generative AI Use

The authors declare the use of generative AI (GenAI) in the research and writing process. According to the GAIDeT (Generative AI Delegation Taxonomy; 2025), the following tasks were delegated to GenAI tools under full human supervision: proofreading and editing, summarizing text, adapting and adjusting emotional tone, and reformatting. The GenAI tools used were Writefull (Writefull) and CoPilot (Microsoft Corporation). Responsibility for the final manuscript lies entirely with the authors. GenAI tools are not listed as authors and do not bear responsibility for the final outcomes.
