# Supplementary material for: A Machine Learning–Guided Lyric Analysis Peer Support Intervention (LAPSI) for Psychological Distress in South African Women: Protocol for an Exploratory Sequential Mixed Methods Study
Source: JMIR Res Protoc. 2026 Oct 21;15:e90426. doi: 10.2196/90426 (PMC13735179; doi:10.2196/90426)
Supplement: Supplementary file 1 [file resprot_v15i1e90426_app1.docx]

**Appendix 1**

***Table 1:*** *Complete search strings and the corresponding boolean logic for scoping review*

| **Electronic Databases** | **Search Strategy/Terms** |
| --- | --- |
| PubMed | (((((((((((((((((((((intervention*) OR (program*)) OR (therapy)) OR (support)) OR (counseling)) OR (treatment)) OR ("Intervention Studies")) OR ("Program Evaluation")) OR ("Health Promotion")) OR ("Psychological Intervention")) OR (pharmacological interventions)) OR (psychological interventions)) OR (psychosocial support)) OR (holistic approaches)) OR (alternative strategies)) OR (mental health strategies)) OR (mental health support)) OR (play therapy)) AND ((((((((((((mental health) OR (psychological distress)) OR (depression)) OR (anxiety)) OR (PTSD)) OR (trauma)) OR (psychosocial stress)) OR (mental illness)) OR ("Mental Health")) OR ("Mental Disorder")) OR ("Depression")) OR ("Anxiety Disorder"))) AND (((((HIV-positive) OR (HIV-negative)) OR (PLWH)) OR ("HIV Infections")) OR ("HIV Seropositivity"))) AND (((((women) OR (female)) OR (gender)) OR ("Women")) OR ("Female"))) AND ((((Sub-Saharan Africa) OR (SSA)) OR (South Africa)) OR ("Africa South of the Sahara") |
| Scopus | TITLE-ABS-KEY((intervention* OR program* OR therapy OR support OR counseling OR treatment OR "Intervention Studies" OR "Program Evaluation" OR "Health Promotion" OR "Psychological Intervention" OR "pharmacological interventions" OR "psychological interventions" OR "psychosocial support" OR "holistic approaches" OR "alternative strategies" OR "mental health strategies" OR "mental health support" OR "play therapy") AND (mental health OR "psychological distress" OR depression OR anxiety OR PTSD OR trauma OR "psychosocial stress" OR "mental illness" OR "Mental Health" OR "Mental Disorder" OR "Depression" OR "Anxiety Disorder") AND ("HIV-positive" OR "HIV-negative" OR pldh OR "HIV Infections" OR "HIV Seropositivity") AND (women OR female OR gender OR "Women" OR "Female") AND ("Sub-Saharan Africa" OR SSA OR "South Africa" OR "Africa South of the Sahara")) |
| Web of Science (WOS) | TS=(intervention* OR program* OR therapy OR support OR counseling OR treatment OR "Intervention Studies" OR "Program Evaluation" OR "Health Promotion" OR "Psychological Intervention" OR "pharmacological interventions" OR "psychological interventions" OR "psychosocial support" OR "holistic approaches" OR "alternative strategies" OR "mental health strategies" OR "mental health support" OR "play therapy") AND TS=(mental health OR "psychological distress" OR depression OR anxiety OR PTSD OR trauma OR "psychosocial stress" OR "mental illness" OR "Mental Health" OR "Mental Disorder" OR "Depression" OR "Anxiety Disorder") AND TS=("HIV-positive" OR "HIV-negative" OR pldh OR "HIV Infections" OR "HIV Seropositivity") AND TS=(women OR female OR gender OR "Women" OR "Female") AND TS=("Sub-Saharan Africa" OR SSA OR "South Africa" OR "Africa South of the Sahara") |
| PsycInfo | ((intervention* OR program* OR therapy OR support OR counseling OR treatment OR "intervention studies" OR "program evaluation" OR "health promotion" OR "psychological intervention" OR "pharmacological interventions" OR "psychological interventions" OR "psychosocial support" OR "holistic approaches" OR "alternative strategies" OR "mental health strategies" OR "mental health support" OR "play therapy") AND ("mental health" OR "psychological distress" OR depression OR anxiety OR PTSD OR trauma OR "psychosocial stress" OR "mental illness" OR "mental disorder" OR "anxiety disorder") AND ("HIV-positive" OR "HIV-negative" OR PLWH OR "HIV infections" OR "HIV seropositivity") AND (women OR female OR gender OR "Women" OR "Female") AND ("Sub-Saharan Africa" OR SSA OR "South Africa" OR "Africa South of the Sahara")) AND PEER(yes) |
| DANS Station | Search Method: Advanced search with metadata filters  Search Terms: Mental health, depression, psychological distress, psychosocial stress, anxiety, interventions, women, HIV, Sub-Saharan Africa  Filters: Dataset type, publication year range |
| Google Scholar | Mental health, depression, anxiety, women OR female, HIV OR AIDS, intervention OR program OR therapy, "Sub-Saharan Africa |
